# The Slit–Robo signalling pathway in nervous system development: a comparative perspective from vertebrates and invertebrates

**DOI:** 10.1098/rsob.250026

**Published:** 2025-07-09

**Authors:** Nicole Sanhueza, Evelyn C. Avilés, Carlos Oliva

**Affiliations:** ^1^ Faculty of Biological Sciences, Pontificia Universidad Católica de Chile, Santiago, Metropolitan Region 8331150, Chile; ^2^ Centro Interdisciplinario de Neurociencia, Pontificia Universidad Católica de Chile, Santiago, Metropolitan Region 8331150, Chile

**Keywords:** Slit–Robo signalling, axonal guidance, neuronal development

## Abstract

During nervous system development, growing axons find their targets with the help of guidance cues. These cues, which can be secreted molecules provided by neighbouring cells or transmembrane proteins mediating cell–cell contacts with the growing axons, act as either chemoattractants or chemorepellents. Over the last decades, several axon guidance molecules have been identified. One of the classical guidance cues is the Slit protein. Slit is a secreted protein, initially identified in a genetic screen in the fruit fly *Drosophila melanogaster* but later shown to be present in other organisms including vertebrates. Slit was originally classified as a repellent guidance cue, but nowadays it is recognized as a promoter of axonal growth in some contexts. Slit action is mediated mainly by the Roundabout (Robo) family of single pass transmembrane proteins, although it has been shown more recently that other proteins can also function as Slit receptors. In this review, we describe the main aspects of Slit–Robo signalling during development of the nervous system. We start with a historical view of the discovery of these proteins, followed by a description of their main molecular characteristics. We then explore specific examples that describe the functions and signal transduction mechanisms of this signalling pathway.

**Figure:**
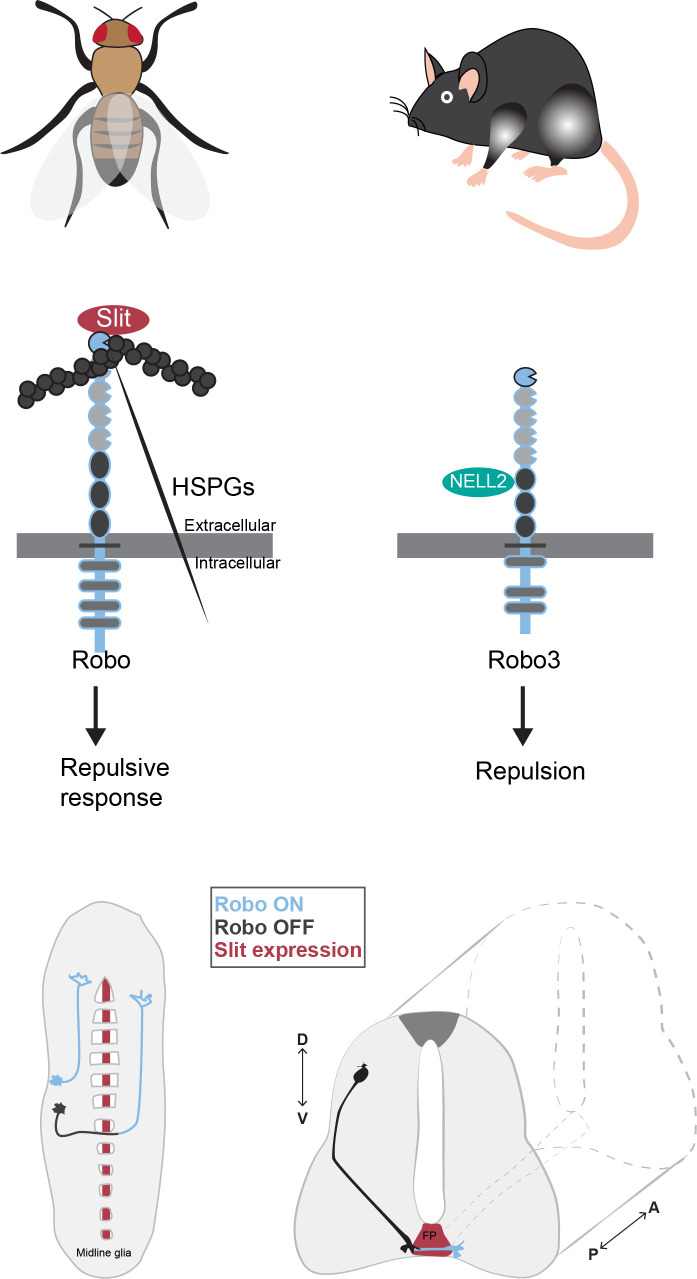

## 1. Introduction

A key event in our comprehension of axonal guidance and cell migration during development came with the discovery of Slit proteins. Slit was first detected in the fly embryonic midline glial cells of the ventral nerve cord (VNC), from where it is released to the extracellular space and distributed along axon tracts. Slit is a member of the protein family containing EGF-like repeats [1]. In vertebrates, similar to cells at the ventral midline of the fly, Slits are secreted from the floor plate of the developing nervous system [2]. In the developing central nervous system (CNS) of the embryo, *sli* mutations cause the normal scaffold of commissural and longitudinal axon tracts to collapse [1,3]. Since their discovery, studies have revealed that Slit proteins are conserved within metazoans, underscoring their crucial roles in nervous system development. Remarkably, Slit proteins serve mostly as repulsive cues for axonal guidance, directing developing axons towards their proper targets and away from undesirable areas. This repulsive activity is mediated by the receptors of the Roundabout (Robo) family, expressed on the surface of navigating axons.

The discovery of Robo came out of a *Drosophila* genetic screen study [4]. *robo* mutants displayed aberrant midline axon crossing phenotypes, which had not been described at the time. Commissural axons recrossed inappropriately several times forming roundabout pathways, hence the name Robo [4,5].

Subsequent research revealed Slit as a ligand for the Robo receptor in both vertebrates and *Drosophila* [2,6]. Since then, Slit and Robo have been shown to exhibit evolutionary conservation across species, sharing their repulsive role in axon guidance from vertebrates to invertebrates [2,6,7].

*slit* and *robo* interact genetically in *Drosophila* [6], whereas *in vitro*, the proteins they encode have been shown to interact biochemically [2,8,9]. Furthermore, it has been demonstrated that Slit can bind Robo at the cell surface [8,10].

## 2. Slit and Robo proteins have conserved domains and present diverse expression patterns

### 2.1. Slit and Robo protein structures

Slit is an extracellular matrix-secreted glycoprotein [3] of approximately 190 kDa [2]. The vertebrate genome encodes three distinct Slit genes: *slit1*, *slit2* and *slit3*, while *Drosophila* has a single *slit* gene [2], same as *Caenorhabditis elegans* that encodes only one *slit* gene (*slt-1*) [11].

**Figure 1.**
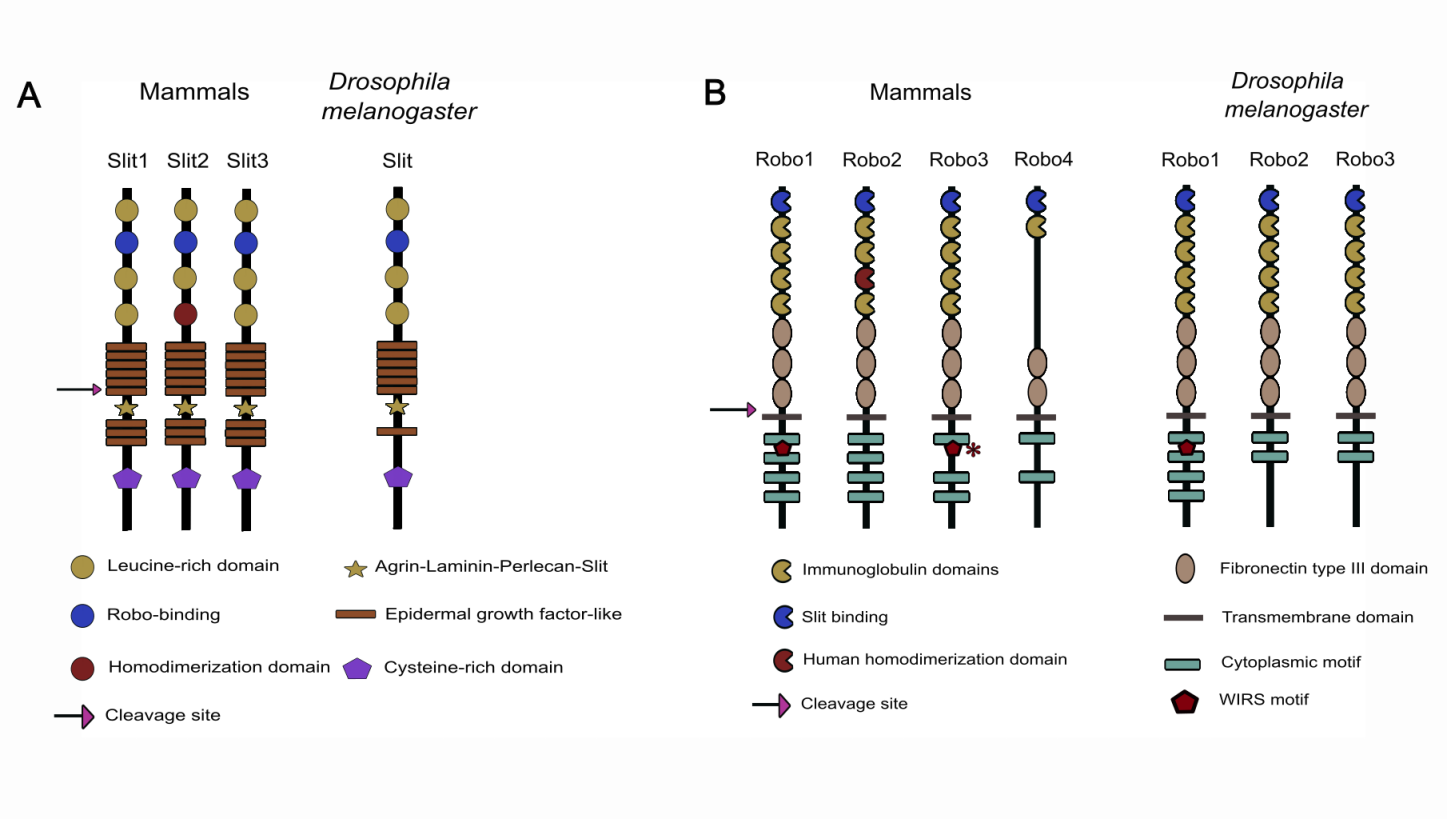
Schematic representation of Slit and Robo structure. (A) Mammals encode three Slit and *Drosophila* only one. Both share a conserved structure containing four Leucine-rich domains (LRR) where the second one serves as the Robo binding site, seven to nine epidermal growth factor-like (EGF) domains with an Agrin–Laminin–Perlecan–Slit (ALPS) motif, and a cysteine-rich domain at its C-terminal. The cleavage site is found between the fifth and sixth EGF domains. (B) Four and three Robo receptors are encoded in mammals and *Drosophila*, respectively. Robo receptors exhibit immunoglobulin domains (Ig) and three Fibronectin (FNIII)-like domains in their ectodomain. Ig4 correspondent to the human homodimerization domain is conserved in hRobo1−3. Its intracellular domain presents from two to four cytoplasmic conserved domains (CC0–CC3). Additionally, Robo1 encodes for WIRS binding motif, while human Robo3 contains a similar consensus sequence that has not yet been experimentally verified (asterisk).

All Slit proteins share a common structural framework (figure 1), consisting of four leucine-rich domains (LRR) (D1–D4); between seven and nine epidermal growth factor-like (EGF-like) domains, in *Drosophila* and vertebrates, respectively [1–3,12,13]; an Agrin–Laminin–Perlecan–Slit (ALPS) conserved spacer motif (also termed Laminin-G-like domain); and a cysteine-rich dimerization domain at the C-terminal end [2,3,14]. Thus, because of their specialized architecture Slit proteins have multiple functions, which enables them to participate in a wide range of signalling processes and protein–protein interactions. Slit proteins undergo cleavage between the fifth and sixth EGF-like domains to form two fragments, the N-terminal (Slit-N) of approximately 150 kDa, predominantly associated with the cell surface, and a short C-terminal segment (Slit-C) of approximately 55–60 kDa [2,15]. The N-terminal fragment is particularly important due to its role in mediating axon guidance. In *Drosophila*, the Robo receptor shows a preference for the D2 domain, within Slit-N, as the major binding site [16]. This interaction is critical for binding to the Robo receptor, which not only initiates the repellent response but also contributes to the precise control of neuronal migration [17,18]. Interestingly, it has been shown that the binding affinity between *Drosophila* Slit and the three Robo receptors is similar [16]. In vertebrates, the Slit2 protein displays an additional layer of complexity through the self-association by its D4 domain, forming stable homodimers [19].

**Figure 2.**
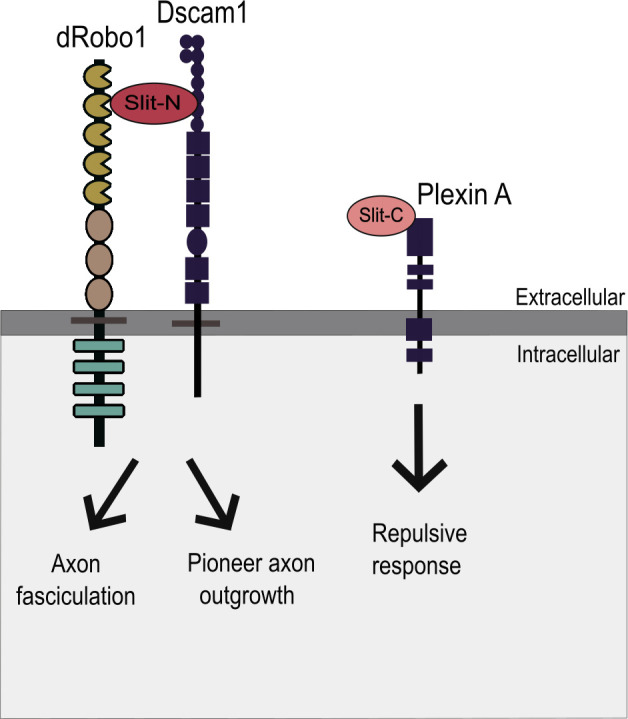
Slit fragments interactions. Information about the specific functions of Slit fragments has only been recently investigated *in vivo*. For example, Slit-N promotes the growth of pioneer axons across segment boundaries in *Drosophila* embryos, which involves its binding to Down syndrome cell adhesion molecule 1 (Dscam1), forming a complex with Robo1 [20]. In addition, Slit and Slit-N can interact with Robo1 to ensure the maintenance of the axons fasciculation post-midline crossing, while both fragments are found associated with the extracellular matrix [21]. On the other hand, the contribution of Slit C-terminal portion to signalling through Robo is yet to be proven [18]. However, Slit-C binds to Plexin A, a Semaphorin receptor in mice [22] involved in guidance of growth cones through the floor plate [23] while it cannot interact with Robo1 [21].

The specific functions of Slit fragments were investigated *in vivo* only recently (figure 2). Although it is generally thought that Slit–Robo signalling is involved only in axonal repulsion, it has been described that in some contexts Slit-N also can promote growth and branching of axons. Indeed, Wang *et al.* showed that the N-terminal fragment of vertebrate Sli2 (Slit2-N) led to axonal growth and branching of sensory neurons of the dorsal root ganglia in an *in vitro* assay [15]. Notably, only the N-terminal fragment but not the full length Sli2 has this activity, in this paper they do not determine the receptor, so this activity may be Robo-independent. This activity of the Slit2-N fragment is context specific, since it does not induce axonal branching of olfactory bulb neurons [24]. Furthermore, the axonal elongation and branching promotion activity is blocked by co-treatment with full length Slit or a Slit uncleavable recombinant version [24]. Consistently, Slit-N promotes the growth of pioneer axons across segment boundaries in *Drosophila* embryos, involving its binding to Down syndrome cell adhesion molecule 1 (Dscam1), and forming a complex with Robo1 [20]. Thus, Slit-N seems to have a conserved role in axonal growth, which needs further investigation. In addition, it is known that Slit and Slit-N can interact with Robo1 to ensure the maintenance of axonal fasciculation post-midline crossing, and both full length Slit and Slit-N are found associated with the extracellular matrix [21]. On the other hand, the contribution of Slit C-terminal portion through Robo signalling is yet to be proven [18]. Slit-C cannot interact with Robo1 [21], but it does bind to Plexin A (figure 2), a Semaphorin receptor in mice [22] involved in the guidance of growth cones through the floor plate [23].

Recently, a Slit metalloprotease able to generate Slit-N and Slit-C was identified. This protein is a member of the BMP1/Tolloid family of Astacin-like metalloproteases and was named Tolkin (Tok; also known as Tolloid-related, Tlr, or Piranha). In *Drosophila*, Tok is responsible for Slit cleavage *in vitro* and *in vivo* [25], thereby modulating its signalling activity specifically in longitudinal axon guidance. Notably, the expression of the Slit-N fragment was sufficient to rescue the *tok* mutant phenotype [25], supporting a critical role of Tok producing the Slit-N fragment, which promotes longitudinal axon growth and fasciculation.

Robo is a single-pass transmembrane receptor belonging to a subfamily of the immunoglobulin (Ig) superfamily proteins, with an apparent molecular weight of approximately 180 kDa for *Drosophila* Robo1 [26,27] and 210 kDa for Rat Robo1 proteins [8]. In mammals, four Robo receptors (Robo1–Robo4) have been identified, three in chick and *Xenopus*, while *Drosophila* has three Robo receptors (Robo1–Robo3), and *Caenorhabditis elegans* only one (SAX-3) [5,28–30]. Robo has an ectodomain with five Ig domains, the first two are the most conserved parts of the extracellular domain [5], and three fibronectin type III (FNIII)-like domains [5,28] (figure 1). Furthermore, Robo bears a transmembrane domain, and two to four Conserved Cytoplasmic (CC0, CC1, CC2 and CC3) motifs in its intracellular tail [5,28]. Additionally, Robo1 has a motif that allows the activation of the Scar/Wave Regulatory Complex (WRC; see below), called WIRS motif (WRC-interacting receptor sequence) between CC0 and CC1 [31,32] which is conserved in other vertebrates and *Drosophila*. Mammalian Robo4, on the other hand, has only two membrane-proximal FNIII domains and two membrane-distal Ig-like domains, thus missing the Ig4−5 and the first FNIII domain [33]. Robo4 also lacks 2 of the 4 cytoplasmic motifs (CC1 and CC3), and even though the characterization of its role in the nervous system is very limited, its expression during brain development has been described (see below). On the other hand, the three *Drosophila* Robo receptors share the same ectodomain structure of five immunoglobulin domains and three FNIII domains. The cytoplasmic domains of the Robo receptors exhibit more divergence. While Robo2 and Robo3 lack the CC2 and CC3 motifs, Robo1 possesses all four of the short, conserved consensus sequences (CC0–CC3) and the WIRS motif [32,34].

The function of the Robo cytoplasmic domain in signalling has been studied in some detail. It is responsible for mediating the repulsive response, as demonstrated using a chimeric receptor consisting of the Robo ectodomain and Frazzled (Fra, a receptor involved in growth cone attraction that responds to the protein Netrin) cytoplasmic domain. In these experiments performed in *Drosophila*, the authors observed that Robo-Fra chimeras switched the repulsive response to attraction [35]. Despite lacking autocatalytic activity, the tyrosine 1040 in *Drosophila* (Y1073 of human Robo1) Robo’s cytoplasmic domain can potentially be phosphorylated by ABL kinase [36] and used in Robo-mediated signal transduction. This tyrosine is contained within the three proline-rich regions located in the CC1 motif [5] that can bind to cytoplasmic adaptor proteins. Several short-conserved regions have been found in the Robo intracellular domain, that are essential for recruiting downstream effectors. Despite the divergence of the Robo cytoplasmic domain structure compared with those described in other guidance receptors involved in axonal repulsion such as Drl and Unc5, experiments with chimeric receptors suggest that all of these receptors converge on a common repulsive pathway involving the protein Trio GEF, a GTP exchange factor that regulates small Rho GTPases activity [37]. Thus, these findings reveal the possibility that the functional differences between repulsive receptors may not lie in the intracellular signalling cascade they trigger, but rather in their extracellular domains and their ability to bind specific ligands.

Regarding the ectodomain, the requirement for the repulsive function of Robo1 Ig1 domain has been highlighted recently [38]. This is probably explained by the fact that Robo binds to the D2 domain of Slit through its Ig1 domain [39,40], which can also bind heparin [41]. Furthermore, based on the crystal structure of the Ig4–Ig5 domains of hRobo2, it was shown that Robo receptors were able to dimerize through the Ig4 domain [42]. By contrast, using the structure of the Ig1–Ig4 region and Ig5 domain of Robo1 ectodomain, another study concluded that Robo1 forms a dimer of dimers at the cell membrane, which is not modified by Slit binding [43]. However, later studies, using the intact hRobo2 ectodomain harbouring Ig1–Ig5 plus the three FNIII domains, confirmed that the Ig4 domain mediates dimerization, and also demonstrated that dimerization is normally blocked by auto-inhibition [44]. In this model (which was suggested to be conserved in other Robo receptors), Slit binding is probably capable of relieving the Robo auto-inhibition state, promoting the release of the Ig4 domain and its dimerization, which is proposed to be required for signalling [44]. Interestingly, the auto-inhibition can be mediated by the second FNIII domain of the same molecule in *cis* and further fastened by an opposed Robo molecule in *trans*, presented on the membrane of a neighbouring cell or a neighbouring cell process [44]. On the other hand, mammalian Robo3 does not bind Slit with high affinity due to specific alterations in its first immunoglobulin domain [45].

In the *Drosophila* VNC, proteolytic processing of the Robo1 receptor is important for its correct signalling, as demonstrated by Coleman *et al*. [46]. According to this model, the metalloprotease Kuzbanian (Kuz), a homologue of the matrix metalloproteinase (MMP) ADAM10, can induce the cleavage of the Robo1 extracellular domain. As shown by Coleman *et al*. [46], this Kuz-mediated cleavage is essential for the Robo1 repulsive response during axonal control at the midline of the *Drosophila* embryo [46], as evidenced by experiments using an uncleavable form of Robo. The creation of an uncleavable form of Robo (Robo-U) through the exchange of the first three FNIII domains of Robo1 for the corresponding domains of Frazzled, and its inability to fully rescue the *robo* mutant phenotype [46], suggests that the region containing these FNIII domains is important for Robo processing and, consequently, for its normal repulsive function. Furthermore, in human cancer cells, Robo1 is sequentially cleaved by metalloproteinases and gamma-secretase, suggesting its involvement in signalling pathways [47]. Whether this processing also occurs in other Robo receptors in *Drosophila* and vertebrates remains to be determined.

### 2.2. Slit and Robo receptors expression patterns in the nervous system

**Figure 3.**
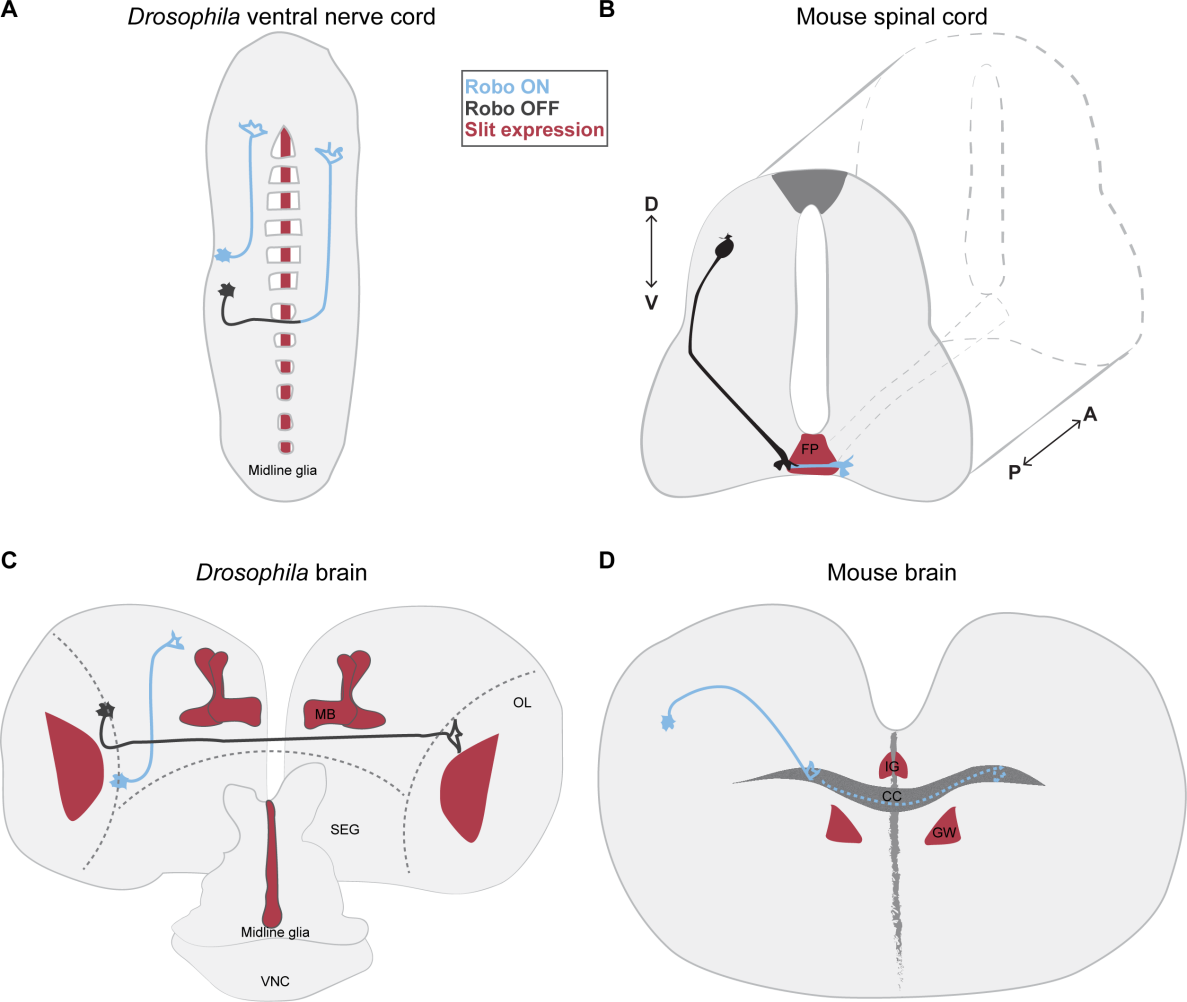
Mechanisms of Slit–Robo-mediated axon guidance in the mouse and fly central nervous system. (A) The fly VNC is the region in which Slit expression and function is best characterized. Slit is secreted by the midline glia and axons will respond to Slit according to the presence of Robo in the membrane of the growth cone. (B) In the mouse spinal cord, pre-crossing commissural axons do not express Robo1/2 (signal OFF) on their surface. However, as they reach the midline, they express Robo1/2 (signal ON) and respond to Slit secreted from the floor plate (FP) glia to exit this structure. P: posterior, A: anterior. (C) In the *Drosophila* larval brain, Slit is expressed in the optic lobe (OL), neuropil, and in the central region is enriched in the mushroom bodies (MB), in the most posterior part of the brain, called the subesophageal ganglion (SEG), Slit expression is observed in the midline glial, as well as in the ventral nerve cord (VNC). No extensive characterization of Slit and Robo function has been performed in the fly brain, but the architecture of some neuronal populations may depend on the activity of Robo receptors and their proximity to Slit sources such as the MB. (D) In the mouse brain, callosal axons display Robo signalling (in blue) to cross through the corpus callosum (CC) to the contralateral side. Indusium griseum (IG) and glial wedge (GW) express the Slit ligand.

Expression patterns of Slit and Robo are dynamic during development and have been investigated in detail in some systems. Here we will give an overview that will be complemented in the next sections with more in-depth analysis for specific examples. In invertebrates such as the fly Slit is secreted from ventral midline cells during embryo development (figure 3A), while in the mouse Slit proteins are primarily released from the floor plate of the developing spinal cord [2,3] (figure 3B). In the *Drosophila* larval and pupal brain, Slit is expressed in the mushroom body (MB), the optic lobe and the most posterior part of the brain, called the subesophageal ganglion (SEG) [48,49]. In these regions Slit is expressed in glial cells and neurons [50,51] (figure 3C). In the adult brain, Slit protein is strongly detected in the MB [49].

Slit distribution in the vertebrate brain is more complex than in the spinal cord, implying more diversified roles in axon guidance and other developmental processes in this region. For instance, in the mouse visual system, only Slit1 and Slit2 are expressed, whereas in zebrafish, Slit2 and Slit3 are both required and cooperate in guiding retinal ganglion cell axons [52–54]. In the mouse brain, the glia of the indusium griseum (IG) and glial wedge (GW) express Slit proteins to regulate the formation of the corpus callosum [55,56] (figure 3D). Interestingly, Slit expression persists in the adult nervous system. In the peripheral nervous system (PNS) and spinal cord of the adult mice, Slit1 expression is more restricted to neurons, while Slit2 and Slit3 display a more widespread expression, including glial cells and fibroblasts [57].

Robo1 and Robo2 proteins in the spinal cord are predominantly expressed in post-crossing axons [58], whereas Robo3 is expressed in axons before midline crossing [58,59]. Robo4 was initially identified as an endothelium-specific member of the family [33,60], but its expression has also been detected in the developing nervous system, specifically in the neocortex, hippocampus and cerebellum of stages E16 to P7 in mice, while the protein has been detected in the neocortex and cerebellum [61]. As in the case of Slit, Robo protein expression is also maintained in the adult nervous system. In the PNS and spinal cord of the adult mouse, Robo1 exhibits a broader expression pattern across various cell types within the PNS. By contrast, Robo2 is primarily restricted to neurons and is exclusively localized to axons in the peripheral nerves. Robo3 expression was not detected in the PNS nor the spinal cord of the adult mouse in this study, while Robo4 mRNA was detected in the sciatic nerve, spinal cord and dorsal root ganglia (DRG) [57].

*Drosophila* Robo paralogues also display differences in their expression patterns. Robo1 is widely expressed in the CNS during the embryonic stage, probably expressed in all neurons. By contrast, Robo2 and Robo3 proteins show a more restricted expression. The regulation and expression of Robo2 have been addressed in some detail. Recent enhancer mapping studies using GAL4 reporter lines have revealed that *robo2* expression arises from the activity of diverse enhancer fragments, due in part to enhancer regions within the large first intron of *robo2* (which has 22 kb) that support its expression [62]. Some enhancers direct expression in different cell types, such as early pioneer neurons, midline glia and lateral longitudinal neurons. Notably, specific enhancers are expressed in the midline glia [62], where *robo2* promotes midline crossing of commissural axons by opposing Slit–Robo1 signalling [63]. Robo2-positive axons marked by some enhancers do not overlap with FasII-positive lateral pathways and, in some cases, precede them temporally, suggesting an early role in axonal scaffold formation [62]. During the larval stage, Robo2 and Robo3 were detected in neuronal populations adjacent to the MB [49,64]. Robo3 is also expressed in R8 photoreceptor cells in the visual system [65]. Furthermore, neurons of the medulla and lobular plate in *Drosophila* express the receptors Robo3 and Robo2, respectively [66,67].

## 3. Regulation of Slit and Robo expression and signalling

### 3.1. Transcriptional, post-transcriptional and post-translational regulation of Slit and Robo

This section provides a general overview of the regulatory mechanisms governing Slit and Robo expression, function and signalling output. Some of these mechanisms are described in more detail, along with specific examples, in the following sections.

During nervous system development, the expression patterns of the Slit and Robo genes are closely regulated. The spatiotemporal expression of Slit and Robo is controlled by transcriptional regulation, which is essential for axonal pathfinding and neuronal migration. This fine regulation ensures that neurons migrate to the correct places and axons are guided to the proper targets. In pontine neurons (PN) along the anteroposterior (AP) axis during mouse hindbrain development, Robo2 is a direct target of Hoxa2, a homeodomain transcription factor of the Hox gene family [68]. Hoxa2 regulates Robo2 expression, which facilitates the repulsive Slit–Robo signalling pathway, guiding appropriate pontine neuron migration and guaranteeing appropriate neural circuit construction [69]. Furthermore, in the spinal cord, alternative splicing generates distinct isoforms of Robo1 and Robo2, each exhibiting varying response strengths to Slit [70]. In the *Drosophila* embryo the homeodomain transcription factor Hb9 acts upstream of Robo2 and Robo3 to regulate axonal guidance. In ventrally projecting motor neurons, Hb9 is required for *robo2* expression, and rescue of Robo2 activity in *hb9* mutants rescues motor axon defects [71]. Moreover, Hb9 requires its conserved repressor domain (Eh) for proper regulation of *robo2*. An Hb9 transgene lacking this domain fails to rescue *robo2* expression in *hb9* mutants effectively [71]. This suggests that Hb9 could promote *robo2* expression indirectly, perhaps by repressing a direct regulator of *robo2*.

Another layer of regulation is the post-transcriptional control. For instance, in the chicken spinal cord, the microRNA miR-92 suppresses Robo1 translation specifically in precrossing commissural axons. miR-92 binds to the 3′ UTR of *Robo1* mRNA, which affects translation but not its stability, therefore miR-92 regulates Robo1 expression in the precrossing CAs and consequently modulates Slit sensitivity [72]. Additionally, post-transcriptional modifications in *slit* and *robo2* mRNA that regulate their translation efficiency have been proposed in *Drosophila* [73,74]. *slit* and *robo2* mRNAs are targets of the 10–11 translocation protein orthologue (dTet), which can hydroxymethylate the transcripts, thereby modulating Slit and Robo expression [74]. In *dtet^null^* mutants, Slit and Robo2 protein levels are decreased and display ventral nerve cord defects like those of *slit* and *robo* mutants [74].

Regarding post-translational regulation, during vertebrate commissural axon midline crossing, a ubiquitin-specific protease 33 (USP33) maintains the stability of Robo1 after its interaction with Slit. USP33 promotes Robo1 deubiquitination, which may prevent degradation of the Robo1 receptor and/or enable its recycling following Slit stimulation [75]. In *Drosophila*, Robo availability in the cell membrane is regulated by Commissureles (Comm) [76] through the modulation of Robo localization and trafficking [77,78] as discussed below. In vertebrates, there is no Comm orthologue, however there are several proteins that play similar roles. Robo3 (also known as Rig-1), whose expression by commissural axons correlates inversely with Slit sensitivity, plays a crucial role in preventing premature responsiveness to Slit-mediated repulsion through Robo1 in mammals, where its removal leads to failure of axonal crossing. However, Robo3 does not downregulate Robo receptor in pre-crossing areas [59]. Additional regulatory proteins that ensure that Slit–Robo signalling is only activated at the correct time and location have been described in the spinal cord (see next section).

### 3.2. Additional receptors and extracellular molecules can regulate Slit–Robo signalling

**Figure 4.**
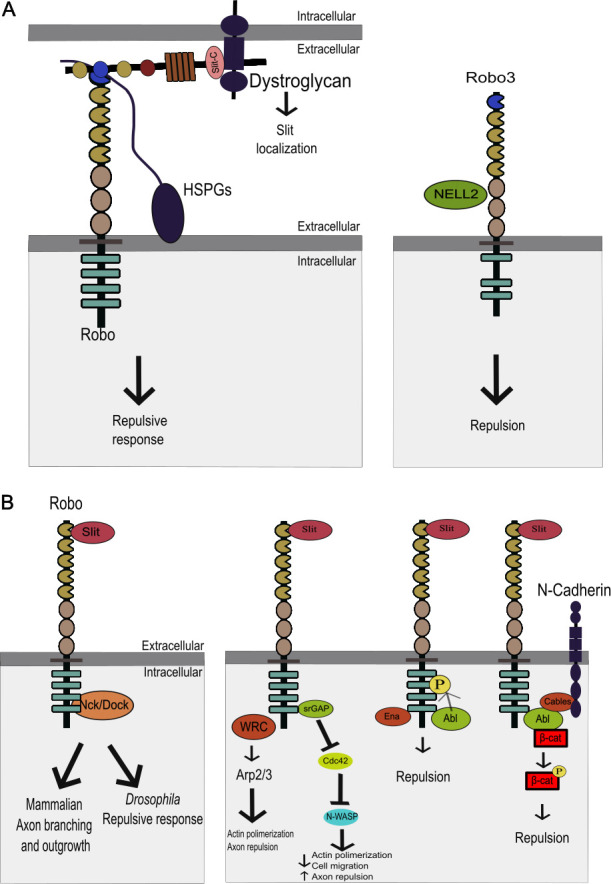
Slit-Robo signalling. (A) The interaction with heparan sulfate proteoglycans (HSPGs) enhances Slit–Robo signalling in both vertebrates and *Drosophila melanogaster* [79,80]. Furthermore, Dystroglycan binds to Slit-C fragment (and possibly to full-length Slit), controlling its distribution in the basement membrane and ensuring its availability for Robo receptors [81]. In mammals, Robo3 receptors interact with ligands such as NELL2, influencing axon repulsion and intracellular signalling pathways [82]. These interactions emphasize the complexity and functional diversity of Slit-mediated signalling during nervous system development. (B) Slit–Robo signalling triggers intracellular events that regulate cytoskeletal dynamics and cellular responses. Upon activation, Robo receptors recruit GTPases, scaffolding proteins and cytoskeletal regulators. Robo internalization via Clathrin-dependent endocytosis is essential for proper signalling. Key downstream events include the modulation of small GTPases like Cdc42, regulated by Robo’s interaction with proteins such as srGAP and Ena, which affect actin dynamics and axon guidance. The recruitment of Nck/Dock proteins facilitates axon branching and repulsion by regulating Rho GTPases. Additionally, Robo activates the Arp2/3 complex and WRC (Scar/Wave Regulatory Complex) to promote actin polymerization at the growth cone, driving axon repulsion. Robo also influences cell adhesion through interactions with N-cadherin, involving the Abl kinase, which phosphorylates β-catenin, leading to its dissociation from N-cadherin and altering cell attachment, ultimately affecting neurite growth.

Slit interacts not only with Robo receptors but also with other receptors, transmembrane proteins and extracellular matrix components, which act as co-receptors or help with its proper extracellular localization. For instance, Slit-C fragment (and probably full-length Slit) can bind to Dystroglycan, a transmembrane glycoprotein, through its laminin-G domain (figure 4A). This binding plays a crucial role in retaining Slit within the basement membrane and the floor plate, supporting its function in axon guidance during spinal cord development [81]. This interaction underscores the importance of Slit extracellular localization in the regulation of neuronal development.

#### 3.2.1. Role of heparan sulfate proteoglycan in Slit and Robo binding

In vertebrates, cell-surface heparan sulfate proteoglycans (HSPGs) play a critical role in enhancing Slit–Robo signalling by binding to the D2 domain of Slit2 [79]. This binding significantly strengthens the interaction between Slit2 and its receptor Robo1, through its Ig1−2 domains [83], thereby promoting the repulsive function essential for cell migration and axon guidance [79,84] (figure 4A). The significance of HSPGs in Slit–Robo signalling is also evident in *Drosophila*, where HSPGs such as Syndecan and Dally-like protein (Dlp) are crucial for the precise localization and effective signalling of Slit within the VNC [80]. Thus, HSPGs, have essential roles in Slit–Robo signalling [80,85].

#### 3.2.2. Slit and Robo proteins have alternative receptors and ligands

In addition to functioning as a ligand–receptor pair, Slit and Robo influence axon development through interactions with additional receptors and ligands, respectively (figures 2 and 4A). It is known that the Slit-C fragment can bind to Plexin A, triggering a repulsive response in the spinal cord [22]. This interaction highlights the crosstalk between Slit–Robo and other guidance cues in the developing nervous system. Slit also binds to receptor protein tyrosine phosphatase 69D (RPTP69D) and Dscam1. Slit enhances the binding of Dscam1 with RPTP69D, which modulates the phosphorylation state of Dscam1 during axonal development in *Drosophila* [86]. Dscam1 dephosphorylation is essential for facilitating axon branch formation, which ultimately increases neural circuit complexity. By influencing Dscam1–RPTP69D interactions and altering the phosphorylation status of Dscam1, Slit introduces a new layer of control to axon growth.

Notably, the mammalian Robo3 does not bind to Slit [45], instead, it binds to the neural epidermal growth factor-like-like 2 (NELL2). This interaction leads to repulsion of commissural axons [82,87] (figure 4A). Interestingly, it was recently shown that Robo3-NELL2 binding becomes stronger under acidic conditions due to the role of the Glu592 residue in Robo3, which acts as a pH sensor [88]. This finding reveals a new way extracellular pH can influence axon guidance and suggests it may also be important for neuronal responses in conditions like ischaemia or certain brain disorders. Additionally, NELL2 could serve as a ligand for Robo2 [89,90]. However, the mechanism and its roles remain poorly understood.

### 3.3. Slit–Robo downstream signalling mechanisms

Upon activation, Slit–Robo signalling initiates a series of intracellular events that can result in cytoskeletal reorganization and other cellular responses (figure 4B). Although Robo receptors lack intrinsic enzymatic activity, they recruit various signalling molecules to mediate downstream signalling. These include cytoskeletal regulators, scaffolding proteins and GTPases that work together to control actin dynamics and cell movement.

It has been shown that Robo1 receptors must be internalized via endocytosis to ensure proper intracellular signalling. Specifically, Chance & Bashaw [91] demonstrated that the repulsive effect of Robo1 in flies requires Clathrin-dependent endocytosis, underscoring the importance of receptor internalization in the control of Slit–Robo signalling. Additionally, endocytosis has also been shown to be important for *Drosophila* Robo3 function during optic lobe development [67]. While this mechanism may be conserved due to sequence similarity among different Robo proteins in various species, experimental evidence for endocytosis of Robo2 in vertebrate and *Drosophila* Robo receptors is not available. During axon guidance and neuronal migration, this dynamic process ensures that signalling events are regulated both spatially and temporally.

Moreover, Slit–Robo regulates neurogenesis in mammalian neural progenitor cells by interacting with the Notch pathway and transcriptionally activating the Notch effector Hes1. However, the activation of Hes1 by Robo1/2’s is independent of Notch signalling and is mediated by the Robo cytoplasmic domains [92], thus, influencing cell fate decisions and neural development. These interactions highlight the versatility of Robo receptors in coordinating various signalling pathways during neural development.

#### 3.3.1. Regulation of small GTPases activity

Small GTPases are GTP-binding proteins that modify the cytoskeleton in order to control cell movement, and they play a central role in transducing signals downstream of activated Robo receptors. The small GTP-binding proteins act as molecular switches, cycling between active GTP-bound and inactive GDP-bound states to regulate various cellular processes, including cytoskeletal dynamics. Robo receptors recruit GTPases and their regulatory proteins, including GEFs (guanine nucleotide exchange factors), Dock/Nck (mammal Nck) and srGAPs (Slit–Robo GTPase activating proteins, in vertebrates) to modulate their activity and downstream signalling pathways. Thus, recruiting RhoGEF proteins downstream of the cytoplasmic Robo domain is required for Slit–Robo signalling [93]. In *Drosophila*, Robo can recruit the adaptor protein Dock to its cytoplasmic domain modulating axonal repulsion [94]. In addition, Robo can bind to Vilse a GAP protein. Vilse regulates Rac activity, and in this way contributes to modulate actin cytoskeleton dynamics and midline repulsion [95,96]. On the other hand, the scaffolding protein Dock/Nck, which binds to Robo receptors in mammals and causes axon branching and outgrowth in cortical neurons *in vitro*, represents a crucial connection between GTPases and the Slit–Robo signalling [97]. Furthermore, Robo is bound by the SH2/SH3 domain present in Nck1 and Nck2 proteins, which mediate Slit-dependent cytoskeleton reorganization that affect the morphology and size of cortical neurons' axons and dendrites [97]. Robo1 can also recruit the WRC to promote Arp2/3 activity, which regulates actin polymerization. This is required for axonal repulsion at the midline in *Drosophila* and mice [31]. These interactions highlight the intricate coordination of signalling molecules and cytoskeletal regulators in Slit–Robo-mediated axon guidance.

The Slit–Robo signalling regulates actin dynamics by modulating the activity of small GTPases and other cytoskeletal regulators proteins. Wong *et al*. [98] discovered that srGAP interacts with the CC3 domain of Robo, affecting Cdc42 activity in vertebrates. Cdc42, a small Rho-GTPase, is involved in actin polymerization at the leading edge of growth cones, crucial for cell motility and axon pathfinding. Slit binding to Robo enhances the interaction between Robo1’s CC3 domain and srGAP1, leading to Cdc42 inactivation [98]. This inactivation inhibits N-WASP and thus the Arp2/3 complex causes actin depolymerization, resulting in axon repulsion and inhibited cell migration [98].

Upon activation, the Robo ectodomain is cleaved and the rest of the protein undergoes internalization. This process seems to be required to activate repulsive signalling by the recruitment of Son of Sevenless (Sos) [91], a Ras/Rho GEF effector that regulates actin organization [99]. This series of events leads to an axonal repulsion response at the midline in *Drosophila* [93].

Another downstream effector of Slit–Robo signalling is Enabled (Ena). The Ena proline-rich protein family that promotes actin polymerization and cell motility. Ena interacts with the Robo receptor to enhance axonal repulsion by stopping the formation and extension of filopodia when responding to Slit [100,101]. Through their interactions with the Robo receptor, Abl and Ena modulate axon guidance in *Drosophila*. While Abl phosphorylates Robo at its cytoplasmic domain to counteract Robo repulsive activity, Ena stimulates repulsion by attaching to Robo cytoplasmic domain and preventing growth cone movement at the midline [36]. By contrast, another study suggested that Abl promotes repulsive midline guidance through its interaction with the *Drosophila* Capulet protein, a member of the cyclase-associated protein (CAP) family [102]. This complexity of mechanisms is due to the different domains of *Drosophila* Abl, which are responsible for controlling both repulsive and attractive axon guidance [103].

#### 3.3.2. Slit–Robo signalling can modulate cell adhesion

Slit–Robo signalling interacts with N-cadherin to regulate cell adhesion and neurite growth. This has been studied in chick retinal cells, where Slit binding to Robo receptors triggers Abl to interact with the intracellular domain (CC3) of Robo, facilitating a connection between the Robo and N-cadherin via the protein Cables a substrate of Abl and CDK5. Upon forming this protein complex, β-catenin is phosphorylated by Abl, leading to its dissociation from N-cadherin. As a result, cell attachment mediated by N-cadherin is compromised and neurites are not formed in cell culture experiments. Furthermore, phosphorylated β-catenin moves into the nucleus, to modify gene expression [104,105]. Similarly, Cables1 connects Robo1-bound Abl kinase to β-catenin during commissural axon guidance, which is important for Wnt-mediated signalling, to steer axons after they cross the midline [106]. Thus, Cables1 is necessary in both situations to link Robo signals to downstream effectors guaranteeing appropriate biological responses.

## 4. Evolutionary conserved role of Slit–Robo in the control of commissure formation

### 4.1. Role of Slit–Robo pathway in the insect ventral nerve cord

The VNC of *Drosophila* is arguably one of the structures that more mechanistic insight has provided regarding the Slit–Robo pathway and a variety of other axon guidance pathways. Here, we will give only a brief overview of the key points; however, readers are invited to explore several excellent reviews that thoroughly discuss the role of these pathways, among others during the development of the VNC [107,108]. The insect VNC is homologous to the vertebrate spinal cord. It is formed by repetitive units named neuromeres [109] and each neuromere hemisegment harbours 36 motoneurons and around 270 interneurons [110–112]. In this structure, the axons can be roughly divided into commissural axons, which cross towards the contralateral side of the VNC crossing the midline; and ipsilateral axons (approximately 10% of the axons), which stay on the side where the cell body is located (figure 3A) [77]. In the midline of the VNC, there is a strip of glial cells (the midline glia) which secretes attractant and repulsive cues that are sensed by axons to choose between ipsilateral and contralateral migration [113].

Slit is the main repulsive guidance cue secreted by the midline glia, in *slit* mutants there is a complete absence of midline repulsion, and all axons stall at the midline. Furthermore, Slit also participates in the development of midline glial cells [3,107,114]. Although *Drosophila* has three Robo paralogues, Robo1 is by far the best studied in this context. In *robo1* mutants, midline axonal tracks display the classic roundabout phenotype, in which many more axons reach the midline and cross it abnormally following a circular trajectory [4,76]. However, this phenotype is different from the *sli* mutant, since only in *robo1robo2* double mutants, the axonal phenotypes resemble those of the *sli* mutation [115]. These results indicate that Robo1 and Robo2 participate in the regulation of midline crossing. Notably, as mentioned previously, all neurons from the CNS express Robo1, although the Robo1 protein is only detected in the longitudinal projections [5,76,116,117]. It has been demonstrated that for commissural axons to cross, it is necessary to downregulate Robo1 in growth cones. This is achieved post-translationally with the help of the protein Comm [77]. An analysis of Comm expression in different neuronal populations revealed that Comm is expressed preferentially in commissural neurons and therefore absent in ipsilateral neurons [77]. On the other hand, in a set of commissural neurons in which the temporal expression pattern of Comm has been determined (RP, drlU, EG and EW neurons), there was downregulation of Comm shortly after the axons crossed the midline [77]. At the mechanistic level, Comm is normally sorted to the late endolysosomal system, and recruits Robo1 to this compartment decreasing its localization in the cell membrane. Interestingly, Comm does not extract Robo1 from the cell membrane; instead, Robo1 is sorted from the *trans* Golgi network to late endosomes and lysosomes where both proteins are degraded [77,78]. When axons have crossed the midline, Comm expression is downregulated and Robo1 is allowed to reach the cell membrane to avoid axons to re-cross the midline.

Nevertheless, Comm is not the only way that Slit and Robo availability can be modulated during the crossing of the midline in *Drosophila*. Manavalan *et al.* showed that Slit undergoes glycosylation in a manner that is dependent on the enzyme Mmy (UDP-GlcNAc diphosphorylase), which is critical for Slit secretion but not for its binding to Robo. In *mmy* mutants, axon tracts defasciculate and shift towards the midline. Furthermore, Robo protein quantity is also affected by *mmy* loss, although by an indirect mechanism [118].

Besides its function in midline crossing, the Slit–Robo pathway is also involved in the positioning of longitudinal axonal tracks in the medio-lateral axis [119]. The other Robo1 paralogues, Robo2 and Robo3, play a principal role in this process. While Robo1 is uniformly expressed throughout the nervous system [5] Robo2 and Robo3 have more restricted expression patterns. Indeed, it was shown that the three Robos define three domains of expression in the axonal tracks at stage 16: the most medial third only expresses Robo1, the intermediate expresses Robo1 and Robo3, while the most lateral third has the three receptors [119]. Loss of function of these proteins leads to displacement of axonal tracks towards the midline, to a degree which depends on the loss of both or each receptor alone. Furthermore, gain of function of these genes in axonal tracks shifts them further away from the midline. Thus, differential expression of the three Robo receptors in the axonal tracks determine the distance from the midline. Although a long range Slit gradient has been proposed to determine the position, no direct experimental evidence supports this model, and several arguments challenge its validity [118,120]. Additionally, it has been recently reported that the function of Robo3 in the positioning of longitudinal pathways is independent of Slit binding [121] by using an edited version of Robo3 lacking the Ig1 domain. Interestingly, using *patched* (*ptc*) mutants that downregulate Slit at a later stage it was shown that Slit plays a post-guidance function in the mature axonal tracks [21]. After axons have reached their targets, Slit is present in axonal tracks, and its absence results in defasciculation, merging and shift of axonal tracks towards the midline. Thus, Slit and Robo play essential roles not only in development but also in maintenance of the nervous system.

### 4.2. Role of the Slit–Robo pathway in vertebrate spinal cord development

In vertebrates, axon guidance molecules have been studied in the commissural neurons of the spinal cord for decades (figure 3B). Of particular interest have been the dI1 commissural neurons whose cell bodies are in the dorsal area of the developing spinal cord and extend their axons ventrally and towards the midline. Upon interaction with the floor plate, a midline structure composed of glial cells, the growing axons change their direction, cross the midline and turn rostrally on the other side. Several signalling molecules and their intracellular pathways are involved in the regulation of midline crossing and have been extensively reviewed in the past [122–124] . It has been long known that Slit–Robo signalling is crucial to prevent these axons from re-entering and re-crossing the midline.

Slit–Robo classically regulates axon midline crossing by a repulsion mechanism, although the specific mechanisms used by the distinct Robo receptors diverge. Slits in the spinal cord are secreted by floor plate cells, which are located ventrally [2,3]. Robo1 and Robo2 respond to Slits and are believed to be active only after the axons have crossed the midline to avoid crossing back to the side where their neuronal somas are. Apart from preventing re-entry into the midline, Robo2 seems to be required for guidance of the post-crossing axons to the ventral funiculus by a diagonal turn after crossing the midline [58], and to prevent the midline crossing of ipsilateral axons [125]. A divergent Robo receptor, Robo3, is expressed in pre-crossing axons and promotes midline crossing by a different mechanism [58,59]. Unlike the other Robo proteins, Robo3 lacks high affinity for Slit, a specific characteristic of the mammalian paralogue [45].

Indeed, loss of Robo3 caused commissural axons to re-route and turn into the longitudinal axis without crossing the midline [58,59]. The importance of Robo3 is underscored by the fact that it has a critical role in the correct formation of commissures in mice, zebrafish and humans [59,126–129]. Although it was initially proposed that Robo3 negatively regulated the repellent activity of Robo1/2 [58,59], the mechanism for this regulation is so far unclear. However, there are results supporting a role in promoting attraction by Netrin1 [45]. Netrin1 induces the phosphorylation of Robo3, which in turn binds to Netrin1 receptor colorectal cancer suppressor (DCC, also known as Frazzled (Fra)) [45].

Robo3 has multiple isoforms. Two of these isoforms [130] are produced through differential intron retention. These isoforms exhibit opposite functions: Robo3.1 promotes midline crossing, while Robo3.2 inhibits it. Moreover, these isoforms are tightly regulated spatially, with Robo3.1 expressed on pre-crossing axons and immediately downregulated as axons arrive to the midline. On the other hand, Robo3.2 is transiently expressed on post-crossing segments [131,132]. However, Robo3.2 is poorly conserved even in mammals. Additionally, Robo3 is the receptor for NELL2, a cue secreted by motor neurons, which contribute to axon repulsion from the motor columns to steer them to the floor plate by promoting Robo3 oligomerization [82,87]. In summary, all Robo signalling pathways directly guide commissural axons both before and after midline crossing through distinct mechanisms.

Slit–Robo signalling is also involved in guidance of motor axons. The spinal cord motor columns house the neurons that innervate the muscles, the motor neurons. These neurons extend their axons from the central nervous system (the spinal cord) to the periphery (muscle) and they exit through specific locations of the spinal cord. Robo2 and Slits mediate the exit of spinal accessory motor neurons through the lateral exit point possibly through a short-range attractive mechanism [133]. On the other hand, Slit–Robo signalling might contribute with its classical repulsive mechanism to guide ventral motor neuron axons [134]. In this case, Robo1 and Robo2 are required together for proper motor axon guidance and fasciculation to the exit site and guidance outside the spinal cord on their way to the dermomyotome by, partially, responding to Slit1 and Slit2. The mechanism is consistent with a repulsive guidance by Robo1/2 that counteracts the attractive action of Netrin1-DCC on motor axons to coordinately direct the proper exit to the periphery [134]. Repulsive Slit–Robo signalling is also required for some cranial motor neuron axon guidance to prevent them from crossing the midline and remain ipsilateral within the hindbrain, both in mice and chicken [135].

Mechanistically, the fine regulation of attractive cues and the repulsive activity of Slit–Robo signalling is required to ensure axons to be attracted to an intermediate target, such as the floor plate at the midline, and once they have reached it to continue their journey away from this structure. A reported mechanism for regulating the timing and location of repulsive activity involves the expression of Robo receptors in axonal growth cones by alternative splicing, discovering a previously unknown layer of regulation [70,131]. Alternative splicing of a specific microexon 6b, included in the Robo1 and Robo2 genes, is controlled by the NOVA splicing factor and regulates the function of these receptors. The inclusion or exclusion of 6b regulates the temporal production of two Robo1/2 isoforms, e6b+ and e6b−, where e6b+ exhibits higher repulsive activity than e6b− [70].

Alternatively, insertion of Robo1 into the cell surface is modulated by RabGDI (Rab Guanine Nucleotide Dissociation Inhibitor) in chick embryos, but the mechanism is different from that of Comm. RabGDI is expressed specifically during midline crossing and promotes Robo1-containing vesicles to be shuttled to the growth cone membrane by Calsyntenin-1 [136–138]. Most recently, like the *Drosophila* Comm gene, the Proline-Rich and Gla Domain 4 (PRRG4) protein was also shown to control the location of hRobo1 receptors on the cell surface [139]. Likewise, the Ndfip1 and Ndfip2 proteins were shown to stimulate the midline crossing of commissural axons in the developing spinal cord, also by controlling surface expression of mammalian Robo1 [140]. In this context, the mechanism is mediated by an interaction with the Nedd4 E3 Ubiquitin ligase to promote the ubiquitylation and degradation of Robo1 [141].

The repulsive role of the Slit–Robo signalling has also been described in axons other than commissural axons. In zebrafish, axons that descend ipsilaterally from the ventral diencephalon are repelled from the midline by Robo2 [142], same as spinal cord ipsilateral axons in mice [125].

### 4.3. A proposed tug of war model between Slit–Robo and Netrin-DCC pathways during midline crossing

There is a well-documented relationship between the Slit–Robo and Netrin-DCC signalling pathways during the development of the nervous system. Netrin is a secreted guidance cue which has been involved in attraction and repulsion of axons depending on the context [143,144]. The attractive effect of Netrin is mediated by its receptor DCC. Slit and Netrin play opposing functions during commissure formation and the positioning of longitudinal axonal tracks along the midline of the *Drosophila* VNC [120,122]. While Robo keeps the tracks away from the midline and in its absence more axons cross the midline, Netrin normally acts as an attractant towards the midline. Even though these functions were initially proposed to be independent [122], further studies in *Drosophila* showed the Frazzled receptor regulates Comm expression independently of Netrin, requiring a different signal. However, the nature of this signal and its source are currently unknown [145,146]. Interestingly, this function is mediated by a Fra intracellular domain that is released upon proteolysis of the full-length protein [147,148]. Thus, the current model in the fly indicates that these two pathways induce attraction and repulsion through independent and interdependent mechanisms and the balance between the two, dictate growth cone behavior at each step.

In mammals, Netrin1-DCC and Slit–Robo signalling pathways also have opposing roles in spinal cord midline crossing. Although, by contrast to the fly, it was originally thought that the action of both pathways was sequential in their common goal to steer axons towards the floor plate (Netrin-DCC) and subsequently avoid re-entry into this intermediate target (Slit–Robo) [149]. A novel idea involving the simultaneous balance of two traditional axon guidance pathways has been proposed, challenging the previous view and revealing that both pathways collide more than previously thought. This idea is consistent with what has been described in the fly VNC [122]. A recent study, using several mutant mice for *in vivo* analysis, claims that pre-crossing axons are indeed responsive to Slit–Robo signalling. However, it is the counteraction of Netrin1-DCC that prevents these axons from being repelled from the floor plate [150]. By generating a series of compound mutant mice for specific alternative splicing isoforms of *DCC* together with mutant alleles for *Robo3*, Dailey-Krempel *et al.* altered the balance between the two pathways and concluded that they interact simultaneously to antagonize each other both during commissural axons entry to and exit from the floor plate [150]. This has been called a tug of war model of interaction between the two pathways and seems to explain the results observed in flies and vertebrates.

### 4.4. Axonal guidance in the *Drosophila* brain

The Slit–Robo pathway plays roles in several regions of the *Drosophila* brain. Unlike the VNC and the spinal cord, there is not a single population of cells that secretes Slit in the anterior portion of the fly brain (figure 3C). The expression pattern of Slit and the three *Drosophila* Robo proteins shows that, in the central brain, Slit is mainly expressed in the MB [48,49], a neuropil involved in learning and memory in adult flies [151,152]. So far only one study has showed that Slit produced in the MB is used by other neurons whose axons are proximal to MB during their development [49]. On the other hand, Slit and Robo proteins are required for the proper development of MB themselves [49,64]. However, since in this study, expression of Robo2 and Robo3 in larval stage was detected in neuronal populations that are adjacent to MB, there is the possibility that the defects observed are due to miswiring of these populations that cannot sense MB Slit, leading to morphological phenotypes in the MB as secondary defects. The participation of Robo proteins has also been studied in the fly antennal lobe [153]. Here, the formation of the antennal commissure and localization of axonal terminals of olfactory neurons is affected in *robo* receptors mutants. While manipulations of Robo and Robo3 mostly affects the position of terminals in specific glomeruli, the manipulation of Robo2 disrupts commissure formation. Slit localization has been identified in the SEG and the midbrain. While neither of these sources is near the antennal lobe, high levels of Slit are present in the midline of the SEG, and this may serve as its the source [153].

Strikingly, sex specific regulation of Robo has been implicated in the formation of sexually dimorphic neuronal circuits [154]. Robo1 knockdown in females promotes male specific neurite formation in mAL interneurons. In this context, Robo1 is regulated by fruitless, a master regulator of male circuits involved in courtship. In the physiological context, FruM, a male specific isoform of this protein represses Robo1 expression to form the ipsilateral projection of mAL neurons that are only found in males.

#### 4.4.1. Visual system in *Drosophila*

The retina of *Drosophila* harbours R-cells that sense visual stimuli towards the optic lobe. The guidance of retinal axons is one of the best studied phenomena in the fly nervous system. R-cells can be divided into R1–R6, which send their axons towards the lamina neuropil, and R7 and R8, which grow further reaching the medulla neuropil [155,156]. Several cell surface proteins have been shown to regulate axonal pathfinding of the R-cells. The optic lobe of the fly (and other insects) is composed of four distinct neuropils: lamina, medulla, lobula and lobula plate. During development, these neuropils are originated from two neurogenic structures, the inner proliferating centre (IPC), which gives rise to the lobula, the lobula complex and the inner medulla, while the outer proliferating centre (OPC) produces the lamina and the outer medulla [157,158]. Robo3 is expressed in R8 neurons, and axonal guidance in these neurons is severely impaired in *robo3* mutants, where axons fail to reach the outer medulla and instead extend to the proximal medulla. Mutant clones in which Robo3 is specifically disrupted in R8 neurons show similar although weaker defects, indicating that Robo3 is autonomously required in these cells to regulate axonal pathfinding [65].

### 4.5. Axon guidance in the vertebrate brain

Similar to the fly brain, in vertebrates Slit distribution in the developing brain is more complex than in the spinal cord (figure 3D), however the molecular mechanisms of axon guidance are conserved. For instance, similar to the mechanism acting on commissural axons of the spinal cord, the action of Robo2 is modulated by Robo3 in dopaminergic and neuroendocrine longitudinal axons from the zebrafish hypothalamus [159]. In mice, Slit–Robo signalling is also required for proper pathfinding of lateral ascending thalamocortical, dopaminergic and serotoninergic axon tracts in the forebrain, and in the hippocampus. Descending corticofugal axons, as well as cortical callosal axons, are also guided abnormally upon loss of Slit–Robo signalling [160–162]. Studies using Slit1/2 double-KO mice suggested that all forebrain tracts (thalamocortical, dopaminergic serotoninergic, corticofugal and cortical) are normally repelled from the ventral midline by Slits [162]. Even though the removal of either one Robo receptor (Robo1 or Robo2) does not cause the same guidance defects as the removal of Slits [160,161], the absence of both receptors results in a phenocopy of the loss of Slit ligands in the forebrain structure, suggesting a cooperative function of Robo1/2 [160]. These results emphasize once again that the repulsive action of the pathway is tightly regulated to operate at the proper time and place, with other molecules probably taking part as well. Indeed, spontaneous Ca^2+^ activity in thalamocortical axons promote the upregulation of Robo1, which is suggested to delay axonal growth in this system [163], consistent with previous findings [161]. Analogous to the commissural axon guidance system, there is interaction between the Netrin-DCC and Slit–Robo pathways in thalamocortical axon guidance. However, in this case, the protein FLRT3 acts as a co-receptor for Robo1, and thus promotes attraction by Netrin via translocation of the DCC receptor to the thalamic axons surface [164].

Slit–Robo mediated repulsion also modulates the pathfinding of axons that project from retinal ganglion cells (RGCs) in the eye to the brain in mammals. Normally, in mice, a relatively large proportion of RGC axons cross the midline to form the optic chiasm, whereas a relatively low proportion of axons remain ipsilateral [165–167]. Of the known Slits, only Slit1 and Slit2 are expressed in the mouse visual system, and the absence of both simultaneously induces profound RGC axon guidance defects. Loss of Slit ligands results in the formation of an ectopic optic chiasm positioned anteriorly to the normal one, which also presents defasciculation. In addition, many ipsilateral RGC axons travel through ectopic locations [54]. Since this phenotype is essentially different from the ones described above, it has been suggested that Slits act as channelling factors defining a permissive zone for RGC axons to extend, rather than as divergence factors [54,168]. Repulsive channelling has also been described in zebrafish, where all the RGC axons cross the midline at the optic chiasm to target the contralateral tectum in the brain [169]. Same as in mice, Slit2 and Slit3, expressed in the visual system, are required and cooperate to guide zebrafish RGC axons during their retino-tectal journey [52,53]. Robo2 is the only Robo receptor located in RGC axons [170], and is indispensable for their guidance to the tectum, as the loss of Robo2 phenocopies Slit2/3 double mutants in the zebrafish chiasm. Similar to Slit loss in the mouse, in the absence of Robo2 (also known as astray), RGC axons are defasciculated and re-cross the midline abnormally [53,168,171]. These observations are consistent with the expression pattern of the ligands and receptors, as well as the repulsive effects of Slits on cultured RGC axons [172–174], while generating an enclosed path for the axons to follow.

## 5. Roles beyond axonal guidance

### 5.1. Slit–Robo signalling coordinate neuronal proliferation and neurogenesis of projection neurons

So far there is little data supporting a function of Slit–Robo signalling in early neurogenesis of the fly CNS. One study has shown that Slit regulates asymmetric division of a population of ganglion mother cell (GMC1) derived from neuroblast NB4.2 in the VNC of the *Drosophila* embryo [175]. Normally, this GMC gives rise to a RP2 neuron and a sibling cell (sib). However, in *sli* mutants GMC1 divides symmetrically to give rise to two RP2 neurons. In this context, Slit promotes the downregulation of two POU domain containing proteins (Nubbin and Mitimere). This finding reveals a non-autonomous function of Slit protein in this process.

By contrast, the function of Slit–Robo has been addressed in several studies using the mouse as a model. During early cortical development, neuroepithelial progenitors in the ventricular zone (apical side) divide symmetrically to increase their number. Later on, excitatory projection neurons are generated by asymmetrical divisions either from apical radial glia cells (direct neurogenesis) or from basal intermediate progenitor cells (indirect neurogenesis) [176,177]. In both cases, newly born neurons migrate along the apico-basal axis on the processes of radial glial cells, while changing their morphology and further differentiating in the cortical plate (basal side) where they settle. Slit–Robo signalling has been implicated in the early processes of cortical development: the proliferation and differentiation of neural progenitor cells. In fact, a balance between proliferation and differentiation has been proposed to be regulated by Slit–Robo signalling. In the absence of Robo1/2 or Slits, cell division in the ventricular zone is inhibited, resulting in a decrease of apical radial glia cell precursors [92]. In parallel, loss of Slit–Robo activity induces abnormal higher production of intermediate progenitor cells, which normally generate from apical radial glia cell precursors [92,178]. These findings are consistent with the discovery that distinct levels of Robo1/2 activity determine the mode of neurogenesis in different species and areas of the central nervous system. Direct neurogenesis (through apical radial glia cells) is associated with high levels of Robo1/2, whereas indirect neurogenesis (through intermediate progenitor cells) in the neocortex is associated with low levels of Robo1/2 [179].

Another aspect of neurogenesis is the process of newly born neurons detaching from the apical side soon after cell division. During retinogenesis, the newborn neurons also detach from the apical (outer) neuroepithelium. In zebrafish, the apical retraction of RGCs depends on Slit1b and Robo3 [180,181] by mechanism that involve the regulation of N-cadherin-mediated cell adhesion [180].

### 5.2. Cell migration and boundaries

#### 5.2.1. Boundary formation in the optic lobe of *Drosophila*

Besides axonal guidance, Slit–Robo is also involved more widely in the architecture of brain neuropils by defining boundaries that limit the localization of different cellular populations. This function has been described in the optic lobe of *Drosophila*. In *robo2, robo3* and *slit* mutants, the morphology of the optic lobe is severely altered [48,51,67]. Notably, in this region of the brain, a localized source of Slit does not appear to be required, as the mutant defect can be reversed by expressing Slit not only in the medulla neuropil but also in other cell populations [48,51]. Furthermore, decreasing the expression of Slit in neuronal or glial cells leads to morphological defects demonstrating that this protein is required in both cell types. Studies investigating the role of Slit in the optic lobe in more specific cell types showed that Slit is required in several cell populations in the inner proliferation centre, including glial cells [50]. In this context, Slit is necessary for proper migration of stem cells towards the IPC, the positioning of cells within this region of the optic lobe, and the wiring of at least one neuronal population that originates from this region during development, specifically, the correct guidance of T4 dendrites [182].

The fly optic lobe shares some similarities with the mammalian cortex, since neuronal migration has been shown to play a role in the assembly of neuronal circuits [66]. Neurons from the GPC region of the optic lobe (named Lawf1 and Lawf2) migrate tangentially and localize between two layers of neurons derived from the OPC and the IPC. Although initially it was thought that only GPC and glial cells expressed Slit, while medulla neurons and IPC-derived neurons expressed Robo3 and Robo2 receptors respectively, as mentioned above subsequent analysis showed that Slit is also expressed in medulla and lobula plate neurons and therefore is co-expressed with Robo3 and Robo2 receptors. In mutants of *slit* and *robo,* GPC neurons are ectopically localized within the tissue, probably due to lack of repulsion among the distinct populations [66]. The same study demonstrates that the Slit–Robo signalling pathway is needed for preventing intermingling between medulla and IPC derived neurons. Additionally, the same group complemented previous findings demonstrating that Net-frazzled signalling is also required to generate borders among optic lobe neuropils [183]. Finally, the co-expression of Slit and Robo receptors in at least some cell populations indicates the existence of an autocrine/paracrine Slit–Robo signalling operating in the optic lobe [67]. In order to better understand how the Slit–Robo pathway regulates optic lobe development, further research is needed to identify downstream effectors and determine whether they are shared with those involved in axon guidance, which so far seems to be the case [67].

#### 5.2.2. Cell migration in the vertebrate nervous system

Slit–Robo signalling regulates neuronal migration through the modulation of focal adhesion kinase (FAK) activity and cytoskeletal dynamics in vertebrates. Slit2 influences Cdc42 activity and lamellipodia formation on *Xenopus* growth cones, therefore affecting cellular protrusions and migration of spinal neurons [184]. Myers *et al.* demonstrated that the expression of a constitutively active FAK disrupts neuronal migration, while Slit2 reduces Cdc42 activity and lamellipodia formation in growth cones, thereby inhibiting cellular protrusions. This regulatory mechanism ensures precise control of cell motility and positioning during neural development.

#### 5.2.3. The role of Slit–Robo signalling in tangential and radial neuronal migration

In vertebrates Slit–Robo has also a role in neuronal migration. Slit was first discovered to influence directional migration of neuroblasts to the olfactory bulb [185]. A similar mechanism has been suggested in humans, where the positioning of neuronal progenitors regulated by Slit–Robo signalling has been proposed as a potential approach to control cell migration for regenerative therapies [185–187]. Moreover, several studies have continued to support the role of Slit–Robo repulsive signalling on several migrating neurons during nervous system development.

Same as the typical commissural neurons in the spinal cord, inferior olive neurons of the brainstem extend axons that cross the midline in route to their target in the contralateral side of the cerebellum. However, their somas do not cross this structure, despite their dorso-ventral migration towards the vicinity of the midline. Migrating inferior olive neurons express Robo3, and in its absence they still form the inferior olive nucleus in its proper location, albeit more slowly and with abnormal cellular shape and polarization [128,188]. Other neurons that project to the cerebellum, such as the pontine neurons, do not reach the midline altogether in the absence of Robo3, although their shapes do not change [45,128]. By contrast, the repulsive action of Robo1 and Robo2, as well as Slit1 and Slit2, is required for proper neuronal migration in the inferior olive, as their somas abnormally cross the midline in the absence of these proteins. Moreover, the inferior olive nucleus develops abnormally in these mutants [189]. The pontine neurons also require repulsive Slit–Robo signalling to migrate in the anterior–posterior phase of their journey. In this system, the expression of the Robo2 receptor is controlled by the transcription factor Hoxa2, and the Slit source is the neighbouring facial motor nucleus [69]. Interestingly, the use of conditional knock out mice showed that the tangential migration of pontine neurons and inferior olive neurons to the midline does not depend on Robo1 and Robo2 in these neurons, nor Slits from the floor plate or facial motor nucleus, but it rather depends on non-cell autonomous mechanisms [190].

In the spinal cord, dI1 neurons migrate slightly in the ventral direction from the dorsal area after their axons cross the midline. Robo2 is enriched in the ipsilateral subpopulation of dI1 neurons. In its absence, the cell bodies of ipsilateral neurons (but not the commissural population) are distributed in regions that are too ventral and/or medial. This effect of Robo2 seems to be a partial result of Slit2 action in the surrounding regions of dI1 neuron migration, since the phenotype in *Slit2^−^*^/−^ mice is less pronounced [125].

Motor neuron cell bodies are contained in the ventral horn of the spinal cord and brainstem. Most motor neurons are born close to their final residence (in the ventral progenitor area), therefore the notion that they do not migrate much. In the spinal cord and brainstem, the repulsive action of Slit–Robo signalling is crucial for motor neurons to reach their proper location, as in Robo1/2 or Slits1/2/3 mutant mice these cell bodies are misplaced in the floor plate, as opposed to be adjacent to it in control animals. This effect seems to be counteracted by Netrin-DCC signalling, suggesting a balance of attractive and repulsive forces to keep the neuronal somas in place [191]. On the other hand, some cranial motor neurons take significant migration courses. The facial branchiomotor motor neurons of the hindbrain take paths divided into segments: a first segment where they migrate posteriorly along the ventral side, and a second segment where they change axis and migrate dorsally within the same area [192,193]. The caudal migration of a subset of facial motor neurons is regulated by Robo1 and Robo2 receptors, as well as the trajectory or their axons. Similar to other systems, facial motor neurons seem to be insensitive to floor plate repulsion in the absence of Robo1/2 [194]. Even though most motor neurons have to avoid the midline and not cross it, the cell bodies of a subpopulation of oculomotor neurons in the midbrain and efferents of the inner ear in the brainstem do cross the ventral midline to settle on the contralateral side. Not much is known about molecular regulation of the latter, but Slit–Robo signalling has been involved in the proper migration of oculomotor somata [192]. Oculomotor neurons express Robo1/2 receptors, which confer sensitivity to repulsive Slit1/2 from the floor plate and thus remain ipsilateral before crossing the midline during early stages. In the absence of Slit1/2 or Robo1/2, oculomotor neurons cross the midline much earlier than normal, suggesting that the ligands and the receptors act in a cooperative fashion to repel the cell bodies from the floor plate at an early stage [195]. Furthermore, the neurons that cross the floor plate present differential Slit2/Robo2 expression and birthdate characteristics with respect to the non-crossing ones [196]. These results underscore that not only the presence or the nature (attractive or repulsive) of a guidance cue is crucial, but also the timing of its availability to define their proper trajectories.

Apart from the active migration that motor neurons need to undergo, it is crucial that these neurons do not migrate outside of the central nervous system into the periphery. Several studies have shown that guidance molecules prevent exit of motor neurons to the periphery by regulating the function of boundary cap cells [197–199]. On the other hand, Slit–Robo repulsive signalling has been implicated in the confinement of mouse and chick motor neurons to the spinal cord through a mechanism that involves an intact basement membrane and does not depend on boundary cap cells [193,200].

Robo1 influences the entrance and the migration of interneurons throughout the cerebral cortex at the stages when these neurons are *en route* to their final position in the mouse embryo [161]. Moreover, in the absence of Robo1, calbindin-positive interneurons invade the striatal region, which is normally devoid of migrating neurons due to Sema molecules that act as repellent [161,201]. Interestingly, the loss of Slit1/2 does not phenocopy the Robo1 mutant defects on tangential migration in this system [202]. It is currently unknown how Robo1 mediates repulsion from the striatum. However, like inferior olive neurons in the brainstem, repulsive Slit1 and Slit2 control the positioning of the cholinergic neurons of the basal magnocellular complex to areas close to the ventral midline, away from the progenitor areas in the basal forebrain [202].

A system that uses guidepost cells to physically narrow and permit the path of thalamic axons *en route* to the cortex has been described [203]. Migrating guidepost cells express Robo1 and Robo2 receptors on their surface, which allow them to respond to the short-range repulsive action of Slit2, both in mouse and in chicken [204]. The correct migration and positioning of the guidepost cells to form a permissive corridor for thalamic axons on which they directionally grow underscores the precise coordination of cellular processes, migration and axon guidance, that have profound consequences on a functional nervous system: the proper synaptic targeting of major projections. Interestingly, these two processes are regulated by Slit–Robo signalling.

In addition to tangential migration of inhibitory neurons, Robo1 and Robo4 play a role in the migration of excitatory pyramidal neurons to the cortex [61,176,205]. After neurogenesis in the ventricular or subventricular zone of the developing neocortex, projection neurons migrate along the radial glial cells to reach the cortical plate, where they will reside. Downregulation of Robo1 in mouse embryos results in delayed migration of newborn neurons on radial glia [205]. Similarly, the downregulation of Robo4 induces migration defects of excitatory neurons to the cortical plate, without affecting their proliferation or differentiation [61]. Slit induces further differentiation or excitatory cortical neurons by stimulating dendritic growth and branching [206]. However, how Slits and Robos interact and transduce a signal remains to be studied in more detail.

### 5.3. Branching and synaptogenesis

#### 5.3.1. Slit–Robo at the forming synapse

We have described so far that Slit–Robo signalling plays crucial roles during multiple aspects of nervous system development, extending beyond its classical role on axon guidance. Neurons form synapses with other neurons or different cell types to maintain the flow of information within a circuit. Unsurprisingly, Slit–Robo signalling has also been involved in synapse formation in vertebrates, although not too much is known about the mechanisms of this process. Morphological changes also occur, as neurons need to arborize and stratify on specific sites. In the zebrafish optic tectum, Slit1a inhibits arborization and premature maturation of axon terminals of retinal ganglion cells, and also reduces presynaptic sites via a Robo2-dependent and independent mechanism [207]. Therefore, Robo2 is both required for retinal axon pathfinding at the optic chiasm (see above) and for proper branching, and negatively regulates synaptogenesis at the final target [171,207]. In mice, Robo2 responds to Slits post-synaptically to mediate the formation of synapses in the CA3 and CA1 neurons, in the hippocampus *in vivo* without affecting arborization [208]. Conditional knockouts for Robo2 in CA1 pyramidal neurons were used to specifically delete Robo2 from post-synapses and in these mice the spine density of proximal CA1 neuron dendrites was reduced. This is specific for excitatory neuron synapses, and Robo2 forms a complex with pre-synaptic Neurexins to establish specificity. The synaptogenic defects observed in Robo2 mutants during development have profound effects on spatial navigation in adult mice, indicating malfunctional properties of the mature circuit in the hippocampus [208]. How to reconcile these two apparent disparate roles of Slit–Robo signalling in synaptogenesis in the zebrafish visual system and the mouse hippocampus? It is possible that there are different systems in place in these two species. Alternatively, there could be distinct mechanisms to promote axonal versus dendrite differentiation. However, even though the net result is different (inhibition or promotion of synaptogenesis) there could be similar cellular or molecular origins that depend on the context, both the presence of other molecules (such as apposed neurexins) or timing.

## 6. Future perspectives

Since the discovery of Slit and Robo proteins in late 1980s and 1990s, we have obtained a substantial amount of data that have allowed us to learn the way axons select their path in detail. In the future, we will probably understand better how complex interactions between this pathway and others involved in axonal pathfinding work together to steer the growth cone and establish the normal wiring patterns.

After the initial discovery of Slit in flies, and later confirmation of an orthologue gene in vertebrates, we have unravelled several molecular details about the way this pathway works in both systems. Interestingly, there are big similarities, not only in the way the signal is transduced but also in the expression patterns. Notably, both models are still actively contributing towards a better understanding of the pathway. One pending task is that some aspects have been assessed only in *Drosophila* or mammals, but confirmation is needed to determine if these aspects are also conserved. For instance, we are lacking studies testing the role of this pathway in neurogenesis in the embryonic nervous system and in synaptogenesis in *Drosophila*, while these aspects have been clearly demonstrated in mice.

A challenge for the future is to identify pharmacological modulators of this pathway, for which screenings using invertebrate models could provide an excellent platform for later validation in mammalian models. Drugs that modulate this pathway could aid in the treatment of neurodevelopmental disorders and cancer, as recent literature increasingly supports the role of Slit and Robo in these diseases.

## Data Availability

This article has no additional data.
